# “We all see things through a different lens based on our life experiences”: co-production of a web-based implementation toolkit with stakeholders across the health and social care system

**DOI:** 10.3389/frhs.2024.1356961

**Published:** 2024-05-15

**Authors:** Cindy Faith Brooks, Susi Lund, David Kryl, Sian Lloyd Jones, Michelle Myall

**Affiliations:** ^1^ National Institute for Health and Care Research Applied Research Collaboration Wessex, University of Southampton, Southampton, United Kingdom; ^2^ School of Health Sciences, University of Southampton, Southampton, United Kingdom; ^3^ The Centre for Effective Services, Dublin, Ireland

**Keywords:** co-production, implementation, toolkit, web-based, stakeholder, innovation, health care, social care

## Abstract

**Background:**

Implementing new innovations across the health and social care system is complex, involving many factors that in recent years have been compounded by Covid-19. While a plethora of implementation tools and frameworks are available, there are limitations in terms of their design and accessibility. Co-production is a valuable mechanism for developing tools that have utility and accessibility for those tasked with using them in health and social care organisations and there is growing acknowledgement of increasing the role of co-production in implementation science. This paper provides novel insight into co-production practices and relevance to implementation science by reporting findings from a study to co-produce a web-based implementation toolkit (WIT) that is accessible, usable and designed to support adaptive implementation across health and social care systems. Key themes relating to the process of co-production are outlined and the value of using co-production in implementation processes are discussed.

**Methods:**

A web-based survey (*n* = 36) was conducted with a range of stakeholders across health and social care. Findings identified a need for WIT. Survey respondents were invited to express interest in becoming part of a co-production group and to take part in three online interactive workshops to co-produce WIT. Workshops took place with the group (*n* = 12) and focused on key developmental stages of WIT.

**Results:**

Online co-production workshops were integral to the development and refinement of WIT. Benefits of using this process identified three interrelated themes: (i) Co-designing key features of the toolkit, (ii) Co-producing a toolkit with utility for users across health and social care settings, (iii) Co-producing a toolkit to support the implementation journey. Our approach of undertaking co-production as a dialogic process enabled generation of these themes. To illuminate discussion of these themes we draw upon iterative co-development of the “active ingredients” of key components (e.g., interactive Implementation Wheel) and functions (e.g., interactive “pop-up” definitions of keyword) and features (e.g., case studies) of WIT.

**Conclusion:**

Using a co-production approach with a range of end-users across health and social care systems, highlights the benefits of understanding implementation processes for users in these settings. User-centred design and processes for ensuring accessibility readily support the translation of implementation into rapidly changing health and social care systems to benefit outcomes for patients, their families, carers, service users and practitioners.

## Introduction

Implementing new innovations or changes to practice across health and social care systems is complex. It requires consideration of a variety of adaptive, multifactorial changes, which have been compounded by Covid-19 (1–7). For example, a qualitative case study approach examined implementation activity by staff employed by Academic Health Science Networks (AHSNs). There are fifteen AHSNs across England who provide the main innovation component of NHS England (5, 6) [now structured as Health innovation Networks (HINs)]. A series of focus groups was conducted with senior and operational staff from across the AHSNs. Participants reported the rapid implementation of innovations, brought about by a number of Covid-19 associated factors. This included changes in NHS governance processes enacted at local and national levels resulting in new processes to enable agile and responsive decision-making alongside an increasing acceptance of risk to manage implementation challenges and an adherence to social distancing regulations which were introduced with minimal notification. Furthermore, a shift to online modalities of working, were reported to improve efficiency of AHSNs, reducing time needed for engagement as well as enhancing inclusivity through reaching a more diverse range of people than would have been possible in person (5).

The rapid implementation of innovations in the context of Covid-19, has highlighted the need for developing implementation tools that are responsive and have utility and accessibility for those tasked with using them in health and social care systems. While numerous implementation tools and frameworks are available (8, 9), limitations can be identified in terms of design, accessibility, and being targeted to specific users (10–15).

There is growing acknowledgement of the role of co-production in implementation science and research and how involvement of users can help to ensure implementation tools and frameworks are accessible, agile and responsive to the needs of those tasked with introducing new innovations or changes to practice in health and social care (16). Involving end-users in co-development is more likely to lead to successful adoption of interventions and changes in practice that bring about improvements in experiences of service users, their families and carers (17, 18).

The concept of co-production has been widely and flexibly used across health and social care research (19). Co-production can be defined as bringing together experts by experience, by occupation and researchers to work together, sharing power and responsibility in an equitable partnership (20). While co-production approaches vary, they share the position that those affected by the research have knowledge and expertise equal to the researchers, making them integral to design and deliver it. In addition, co-production approaches foster two-way learning between researchers and experts by experience and occupation, and can increase experts' sense of self-confidence, empowerment and evidence-based knowledge (21).

Despite increasing recognition of the value of co-production in implementation, there has been limited understanding of the methods involved in applying co-production in practice (22). To address this, we provide methodological insight into co-production in practice through describing the development of a web-based implementation toolkit (WIT) (23).

We approach co-production as a dialogic process, involving activities to elicit reflection, discussion and refinement. Methodological insight is illuminated by examples depicting iterative co-development of the “active ingredients” meaning the key components, functions and features, of WIT through three online co-production workshops. These components include co-development of an *interactive Implementation Wheel*, as well as refinement of an existing *Implementation Checklist*. Co-development further informed key functions of the toolkit including interactive “*pop-up” definitions of keywords, drop-down question and answer style interactive menus* and key features including *embedded examples of implementation* and *case studies* designed to assist implementation in practice. Opportunities and challenges in online co-production are reported.

An Implementation Checklist was initially designed and developed in 2020 by the National Institute for Health and Care Research Applied Research Collaboration Wessex (NIHR ARC Wessex) Implementation Team to help ARC Wessex researchers think about implementation considerations from the outset of their projects. The Checklist was informed by the Medical Research Council Framework for Developing and Evaluating Complex Interventions (24), and the empirically based knowledge and experiences of implementation of the Implementation Team. It was also informed by feedback and consultation with NIHR ARC Wessex research teams and regionally with other ARC Wessex Network members.

The Implementation Checklist comprises of six domains (Table 1), with corresponding statements to prompt the user into considering various factors to support decision-making about implementation. It asks users to indicate whether they agree/disagree with statements and to provide evidence to support their response detailing how these areas are being addressed. Use of the Checklist was encouraged at the inception and throughout a project to support users on their implementation journey and address any challenges which may arise. It was initially developed for use by a variety of stakeholders including: clinical and non-clinical academics, researchers, clinicians, public contributors and managers. Early feedback from users suggested the Checklist prompted thinking about identifying implementation considerations for individual projects, but the format was “clunky” and time-consuming to complete suggesting improvements were required to ensure usability, accessibility and utility.

**Table 1:** Six implementation domains with definitions.

Domain	Definition
Project outputs	This domain encourages considering the deliverables or what is to be produced as a result of a project
Buy-in and engagement	This domain focuses upon who needs to be engaged as part of the implementation process, what routes to engagement to use and how engagement will be maintained during implementation
Fit with health and social care systems	This domain concentrates on how implementation of a project output(s) fit with the changing needs of health and social care systems and local, regional and national directives and policy
Alignment with health and social care priorities	This domain focuses upon how implementation of project outputs(s) aligns with the changing needs of health and social care priorities in local, regional and national directives and policy
Outcomes and impact	This domain enables consideration of the outcomes and impact of a project output(s) for patients, service users, health and social care professionals, third sector organisation professionals and health and social care systems
Adoption and spread	This domain encourages consideration of factors that may influence the uptake of the project output within the original context in which it is to be introduced and to other organisations

**Figure 1:**
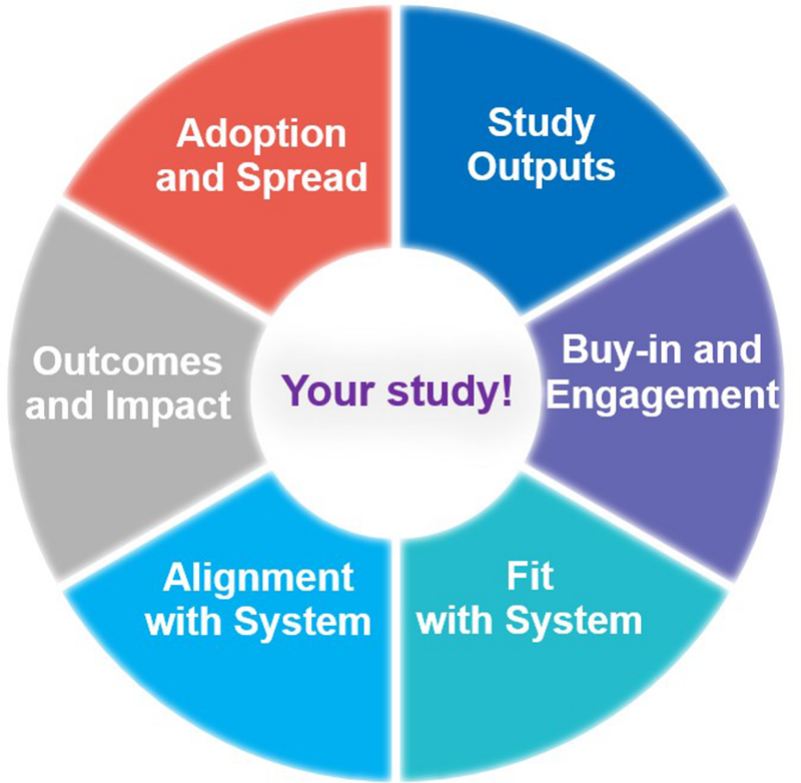
Implementation wheel with six domains.

In 2021, the ARC Wessex Implementation Team delivered a series of four bite-sized webinars aimed at ARC trainees and implementation champions. One session explored “Factors affecting Implementation,” based on the six domains of the Implementation Checklist. To present the Implementation Checklist visually and address the existing identified challenges including usability, accessibility and utility, the Implementation Checklist was redesigned, so all six domains could be visualised in a wheel format in one diagram (Figure 1), with component segments representing each domain. The webinar session was well received and confirmed a need to develop tools that are user-friendly and easily applied by researchers. Discussions with colleagues in other ARCs showed that similar challenges were identified with existing implementation materials, in terms of a need for usable and accessible tools. The Implementation Wheel was presented and used as a visual prompt and a core component in a workshop activity at an ARC Wessex Stakeholder Event in 2022. Feedback from the event indicated that participants from across health and social care considered the wheel to be helpful and usable.

Recognising the potential collective value and utility of the Implementation Checklist, Wheel and Webinars to support a variety of users in their implementation journey, we applied and were awarded funding from the NHS England National Insights Prioritisation Programme (NIPP), to co-produce an implementation toolkit.

This paper reports on findings from a study to co-produce WIT- a Web-based Implementation Toolkit designed to be accessible, usable and to support adaptive implementation across health and social care systems. The study aimed to (i) identify awareness and understanding of implementation toolkits and frameworks by clinical and non-clinical academics, researchers, clinicians, patient and public involvement (PPI) contributors, managers, and third sector organisation representatives in regional networks as well as (ii) establish a co-production group to co-develop the “active ingredients” of WIT comprising representatives from the above groups and (iii) finalise the prototype WIT and its constituent components for testing and evaluation in real world settings. Patient and public involvement (PPI) was essential and integral to these aims so as to ensure development of an accessible and appropriate toolkit so that those who are in receipt of the results of new innovations and interventions are involved throughout the process. Additionally, from the breadth of diverse experience PPI contributors bring and through challenging of any pre-assumptions brought by professionals (Table 2).

**Table 2:** Application of GRIPP 2 reporting checklist (short) (25).

Section and topic	Section and topic	Item	Reported on page number
1	Aim	The aim of PPI in the study was to (1) ensure development of an accessible and appropriate toolkit that has relevance for diverse end-users (2) challenge professional language and assumed knowledge and (3) achieve an inclusive and transparent process.	Pages 3 and 4
2	Methods	A PPI network was approached, and participants invited to take part in an online survey who had an interest in implementation across health and social care settings. PPI representatives were included in the survey to ensure representation and inclusion throughout the whole research process. Interested participants were then invited to take part in online co-production workshops to develop the toolkit.	Page 4
3	Study results	PPI represented 29% of roles in the survey and 29% of roles in the workshop. PPI involvement illuminated key features to be considered and developed including design and accessibility, applicability and general development of the toolkit. PPI ensured clarity and consideration of different interpretations of terminology. They enhanced the toolkit through bringing experience from implementation offering suggestions for improvements by providing examples.	Page 4 Pages 5–12
4	Discussion and conclusions	PPI participants remained focussed on the key aims of the toolkit and played a key role in ensuring other group members did not become distracted or go off at a tangent that risked diverting from the task in hand. PPI members also brought a different lens to enhancing usability by challenging assumptions and taken-for-granted meanings associated with specialist terminology. The importance of considering how PPI are recruited and able to participate including their access to resources such as the internet is essential. The research team facilitated workshops to ensure inclusivity and provided different options to participate including verbal and written at the time of workshops and in between via email.	Pages 12–15 Pages 5–12 Page 7 and Table 3
5	Reflections/critical perspective	PPI brought a breadth of diverse knowledge and experience to the research process and the toolkit development. PPI involvement was highly valued, and their input was recognised and acknowledged by ensuring they were reimbursed for their time and having the opportunity to be included in dissemination activities, such as co-authoring papers.	Page 8, Pages 12–14

## Methods

### Design

There were two key stages in the co-production of WIT. First, following feedback from the stakeholder event to further establish the need for WIT, we undertook a web-based survey conducted with a range of stakeholders (aim i). Second, upon establishing this need, co-producing the “active ingredients” of WIT and a prototype through three online interactive workshops (aims ii and iii).

#### Co-production process

Sharing data alongside the study aims, informed the focus of the workshops. These included considerations of the three key themes including, design and accessibility, applicability of the toolkit for a wide range of users and supporting users in their implementation journey, which were incorporated within the three workshops. Each workshop focused on key developmental stages of WIT. Before each workshop, a programme was sent by email to the group. Details of the programme are provided in Table 3. This included details of the focus of the workshop as well as some questions/considerations to be discussed at the workshop.

**Table 3:** Structure of workshop sessions.

Workshop	Focus
Pre-workshop 1	Research team emailed a copy of the original Implementation Checklist to the co-production group and requested them to feedback their overall perceptions at Workshop 1
1	**Welcome, introductions and aims** • Introductions • Background including why we are undertaking the project • Agree terms of reference for the group • Overview of Project aims **Presentation of survey results-what you told us** **Introducing the ARC implementation checklist and wheel** • What works well? • What doesn’t work so well? • Is there anything, missing? **What do you need from an online Implementation Toolkit?** What is an online Implementation Toolkit? • What implementation topics would be useful to address: ◦ How can public contributors inform implementation? • What does a toolkit need to include to help you know **how** to implement? • What would be useful in terms of design? • What would be useful in terms of access? **Closing remarks/questions and Date of next workshop**
Between workshop 1 and 2	• Research team emailed co-production group to ask them to consider any examples of implementation which had worked well/not worked well at Workshop 2
2	**Welcome, Overview and Focus of Workshop 2** **Introduction and aim of Implementation Toolkit Project** • Introduction and aim of Implementation Toolkit Project • Aim of the workshops and how we will work together **Summary of main discussion points from Workshop 1** • Definitions • How does the Implementation Toolkit add value? • How will the Implementation Toolkit work? • How will the Implementation Toolkit be easily accessible? • Implementation Checklist • Specific areas which we would welcome your feedback on today **Feedback Session 1: Implementation Wheel Update and discussion** • What do you think of the proposals for how the Implementation Wheel will be used in the toolkit? • Are there any other areas that it would be useful to have links to on the home page? **Feedback Session 2: Sharing examples of implementation in practice to help develop case studies for the Implementation Toolkit** • Please share an example of where implementation: ◦ Has worked well in practice ◦ Has not worked well in practice • Next steps, closing remarks/questions
Between workshop 2 and 3	Co-produced feedback from Workshops 1 and 2 about the “active ingredients” of WIT, i.e., the key components (e.g., Interactive Implementation Wheel and refinement of an Implementation Checklist), functions (e.g., “pop-up” definitions of keywords, drop-down question and answer style interactive menus) and features (e.g., embedded examples of implementation and case studies), were shared with the web designer ahead of Workshop 3. The web designer implemented the feedback into the operationalisation of the prototype toolkit. The prototype toolkit was shared by the research team with the co-production group both ahead of and during Workshop 3, within which participants had the opportunity to feedback.
3	**Welcome, Overview and Focus of Workshop 3** **Introduction and aim of Implementation Toolkit Project** • Welcome and overview of the Implementation Toolkit Project • Overview of the session **Summary of main discussion and development areas since Workshop 2** • Development of Web-based Implementation Toolkit • -home page • -domain pages • -case studies/examples • Accessibility considerations • Specific areas which we would welcome your feedback on today **Sharing of WIT prototype (during Workshop 3)** **Feedback Session: Web-based Implementation Toolkit: Design, content and applicability** • What do you think of the Web-based Implementation Toolkit? ◦ Landing page ◦ Domain page ◦ Do you have any thoughts at this stage how you may use the Implementation Toolkit? • Is there anything missing? Next steps, closing remarks/questions • Potential interest in involvement in co-production paper
Post-workshops	Finalisation of WIT Following Workshop 3, any feedback was fed back to the web-designer and the prototype toolkit was finalised. The finalised toolkit was also shared via email with the co-production group following Workshop 3 and no further amendments made.

A dialogic approach involving a combination of activities was used to elicit participant reflection and discussion including MS PowerPoint© to depict visual discussion points and design images, open discussions, as well as using the Zoom chat function (Table 3). Co-production group members were also invited to review and share feedback on the evolving WIT content and design features in-between workshops via email.

### Data collection

#### Web-based survey: establishing the need for WIT

In order to gain understanding of respondents' awareness, understanding and needs regarding implementation and identify awareness and understanding of implementation toolkits and frameworks a web-based survey using MS Forms© was shared via administrators across two NIHR Infrastructure mailing lists including (i) a public involvement network, (ii) a network comprising clinical and non-clinical academics, researchers, managers and third sector organisation representatives. The email accompanying the survey, was addressed to those with an interest in implementation or tasked with implementation in a health and social care setting. Through the public involvement network, patient and public involvement (PPI) representatives were invited to take part in the survey to share their implementation experiences and ensure representation and inclusion throughout the whole research process (Table 2).

At the end of the survey, these self-selecting respondents were asked to indicate if they were interested in becoming a member of a co-production group which involved participating in three online interactive workshops to co-produce WIT. Potential respondents were informed that each workshop would take no more than two hours and that a briefing document and materials relating to each one would be circulated by email at least one and a half weeks in advance of each session.

#### Online co-production workshops: co-producing the web-based implementation toolkit

Over eight months, three two-hour online workshops with the co-production group (*n* = 12) were held via Zoom and facilitated by the research team using a dialogic approach. Participants were encouraged to keep their camera on though this was not mandatory but had been found by the facilitators to aid discussion in previous online workshops.

### Sample

A total of thirty-six respondents completed the survey. Of the thirty-six respondents, roles included Academic (39%); PPI representatives (29%); Clinical Academic (11%); Clinician (8%); Programme Management (5%); Charity (5%); Research and Engineering (3%), with representation across university (47%) primary care (9%); secondary care (16%); patient and public involvement (24%); third sector (4%). Of the thirty-six survey respondents, fourteen participants consented to take part in the online co-production workshops, of which two could not attend due to other commitments at the time of the workshops. Twelve participants formed the co-production group, roles included Academic (43%); PPI representatives (29%); Programme Management (7%); Charity (14%); Research and Engineering (7%) with representation across university (43%), secondary care (14%), third sector (14%) and PPI (29%). Each participant took part in at least two workshops. PPI participants were vital to the study to challenge assumptions, which informed both toolkit design and content and helped to ensure its accessibility to diverse end-users.

### Data analysis

#### Analysis of web-based survey

Open ended survey responses were analysed using the constant comparative method (26). Themes are described in turn. For quantitative data, data analysis involving descriptive statistics which summarised the characteristics of the data was conducted using MS Excel©.

#### Analysis of online co-production workshops

The three members of the research team all attended and facilitated the workshops and two of the researchers took notes during each of the workshops to ensure inclusion of key points. Workshops were audio recorded (with consent) and transcribed by a professional transcriber. In all workshops, researchers relayed key discussion points back to the group to ensure they had understood what participants had said correctly. Summary points of key discussions from the previous workshop were communicated at the start of Workshops 2 and 3 (Table 3). Participants were provided with an opportunity to feedback further comments via email between workshops. This enabled participants to have several opportunities to reflect and provide feedback for refining of the toolkit.

The research team held data analysis meetings following each workshop in which key areas for the development of the toolkit were agreed. To improve the rigour of the analysis and trustworthiness, triangulation of data was performed whereby all researchers independently analysed transcripts with the notes and came together in the analysis meetings to discuss and arrive at a consensus. A thematic approach (27) was used to guide analysis of transcriptions which were used in conjunction with the notes using MS Word©. This involved six core stages; (1) Familiarisation, (2) Identification of coding categories, (3) Grouping codes into themes, (4) Reviewing themes, (5) Naming and refining themes and (6) Presenting the findings. Data workshops were held within the research team to discuss findings and interpretation of responses. Core themes are presented in Table 4.

**Table 4:** Key qualitative themes from the web-based survey and online co-production workshops.

Top three qualitative themes		Open-ended survey questions (*n* = 5)	Total no of items (*n* = 146)	Total no of respondents (*n* = 36)
QT1	Design and accessibility	• What would encourage you to use a web-based online implementation resource? • What would discourage you to use a web-based online implementation resource? • In developing a web-based online resource to support implementation what would be helpful to consider? • Please tell us anything else you think is important to consider when developing the web-based online implementation resource	60 (41%)	31 (86%)
QT2	Applicability for a wide range of users	• In developing a web-based online resource to support implementation what would be helpful to consider? • What would encourage you to use a web-based online implementation resource? • What would discourage you to use a web-based online implementation resource? • List up to three ways a web-based online implementation resource may help you • Please tell us anything else you think is important to consider when developing the web-based online implementation resource	32 (22%)	25 (69%)
QT3	Supporting the implementation journey	• What would encourage you to use a web-based online implementation resource? • What would discourage you to use a web-based online implementation resource? • In developing a web-based online resource to support implementation what would be helpful to consider? • Please tell us anything else you think is important to consider when developing a web-based resource, such as a website? • List up to three ways a web-based online implementation resource may help you	24 (16%)	20 (56%)

## Results

### Web-based survey

#### Qualitative analysis

The web-based survey analysed participants responses to five open-ended questions about what would be helpful to consider when developing a web-based implementation resource, what would encourage/discourage use of a web-based implementation resource, listing up to three ways a web-based implementation resource may help and any other considerations in developing a web-based implementation resource.

Analysis of qualitative responses from the survey revealed three principal themes; (i) design and accessibility; (ii) applicability for a wide range of users; (iii) supporting the implementation journey (Table 5). Themes are described in turn and illustrated through free text extracts. The extracts are labelled according to the participant's role and setting.

**Table 5:** Top three qualitative themes from the web-based survey.

Web-based survey themes		Co-production workshops themes with examples of WIT “active ingredients” produced
QT1	**Design and accessibility**	Co-designing key components of the toolkit Example: • Implementation Wheel component • Refinement of the Implementation Checklist
QT2	**Applicability for a wide range of users**	Co-producing a toolkit with utility for users across health and social care settings Example: • Pop up definition function • Drop down question and answer function
QT3	**Supporting the implementation journey**	Co-producing a toolkit to support the implementation journey Example: • Case studies feature • Examples of implementation feature

#### Design and accessibility

Design and accessibility, including being freely available in the public domain, were reported by respondents as key factors that would encourage use of WIT. An engaging visual design with a clear and uncluttered layout, effective use of colour schemes, simple language with definitions of relevant terminology appropriately placed, as well as signposting to relevant sections, were given as examples to support accessibility and ease of use:

*It would have to be accessible, engaging and enable me to find what I need very easily. The language used within the website to describe the options available would have to match the language I use to describe the topics. It would need to be reasonably simple.* (PPI representative and Charity lead, Third sector)

*It's often good to have an easy read format, and to be succinct, but it can be helpful to have more info[rmation] embedded (maybe by drilling through the top layer page so as not to clutter the first page) for those users who want or need to find out more. Infographics can sometimes be helpful to convey meaning without using a lot of words. Consideration of colour schemes for those with visual impairment and not having things too cluttered for those who can become over stimulated. Keeping terminology suitable for the readership making sure terms are defined somewhere if they are needed… If a website needs to be used by a wide audience, it helps not to have assumed knowledge for using it, but for it to be easy to navigate past the basics for those who don't need the basics.* (PPI representative)

#### Applicability for a wide range of users

An implementation toolkit that has applicability and relevance to a variety of users was considered important by respondents. Suggestions of how relevance could be achieved included giving evidence-based examples relating to different implementation challenges and solutions to “bring them to life.” Other suggestions involved providing a telescopic-style approach to information provision; an overview of key issues and areas for the user to browse, with an option to “deep dive” further information if needed, and flexibility to use the toolkit in accordance with individual and shared needs with colleagues and wider networks in different settings. Public contributors brought a different lens to enhancing usability by challenging assumptions and taken-for-granted meanings associated with specialist terminology and added insights which otherwise may have gone unnoticed:

*We all see things through a different lens based on our life experiences and education. Public contributors bring a fresh perspective to areas being considered. They are not as familiar with areas being looked at, less familiar with the jargon often involved, so can ask the obvious questions which others [may] miss. They can help understanding about how those outside the projects can view subjects being considered.* (PPI representative)

*Some areas of quick content with [the] option to dive more deeply where needed* (Programme Manager, Secondary care)

*Easy to navigate and flow to the format. Content should be evidence informed and include examples to bring it to life.* (Charity Researcher, Third sector)

#### Supporting the implementation journey

Respondents reported the value of an implementation toolkit to enable utility to navigate and chart their implementation journey, providing guidance from initial stages of preparation, across different levels of implementation (e.g., strategic or clinical levels) through to potential challenges and how to address them. Similarly, the provision of a holistic one-stop place for all implementation considerations, whilst simultaneously enabling flexibility to focus on specific areas, was recognised as important:

*I'm hoping you will develop something that guides people through the process, thinking about how they need to prepare for and conduct the implementation to address key barriers that can come up with working with each stakeholder (e.g., those at high strategic level within organisations like the NHS, those within clinical roles on the ground who would be closer to implementation)… things that would be helpful.* (Academic Researcher, University)

*There are so many ways to implement research outputs, it would be great to have something that can draw this into one place… It could be used as part of education and knowledge for people who start within our team. It can be used to share with researchers who should consider implementation as part of grant applications to give them an idea of how they can plan for implementing their research.* (Charity Researcher, Third sector)

*If it was easy to use and flexible to local/project needs. If it gave hints to tackle tricky implementation pitfalls. If it was a tool that I could use to demonstrate progress on implementation or describe barriers to a wide range of stakeholders [in an] understandable way that could then be addressed in a logical and systematic way. If the tool could help demonstrate the impact of good implementation processes which might feel cumbersome or irrelevant to stakeholders.* (Programme Manager, Secondary care)

#### Quantitative analysis

In addition to the qualitative thematic findings, over half of respondents reported having “some knowledge” of implementation (53%); some reporting “a little” (30%). Only 11% stated they had “quite a lot” of knowledge and 1% “very much”.

### Online co-production workshops

#### Qualitative analysis

We approach co-production as a dialogic process, involving activities to elicit reflection, discussion and refinement. Methodological insight is illuminated by examples depicting iterative co-development of the “active ingredients”.

Undertaking co-production as a dialogic process involving reflection, discussion and refinement (Table 3), enabled generation of three key themes: (i) Co-designing key features of the toolkit; (ii) Co-producing a toolkit with utility for users across health and social care settings; (iii) Co-producing a toolkit to support the implementation journey (Table 4). To illustrate discussion of the themes, we draw upon iterative co-development of the “active ingredients” of the key components (e.g., interactive Implementation Wheel) and functions (e.g., interactive “pop-up” definitions of keywords) and features (e.g., case studies). Interwoven is reference to the process of discussions, reflections and refinement involved in this co-production throughout and between workshops. Themes are described in turn and illustrated through verbatim extracts. The extracts are labelled according to the type of participant.

#### Co-designing key features of the toolkit

The use of design including style, images, diagrams and colour schemes featured strongly in discussions surrounding accessibility and were most evident in the co-development of the core component and end-product of the Implementation Wheel during the workshops. Through the co-development process and as will be demonstrated through reference to the process of discussions, reflection and refinements, the Implementation Wheel co-developed from a visual image depicting holistic oversight of the six domains in Workshop 1, through to an interactive online tool by Workshop 3, with functionality to navigate across WIT as a whole. The other core components of the Implementation Checklist and Implementation Webinars were also discussed though they did not feature as consistently and prominently as the wheel in discussions relating to this theme.

Ahead of Workshop 1, a copy of the original Implementation Checklist was circulated to the co-production group who were asked to feedback their overall perceptions on the day. Providing an opportunity for participants to share their views and suggestions provided confirmation to the researchers of the comprehensiveness of the checklist in enabling implementation considerations to be comprehended. Similar to feedback on the early formulation of the checklist discussed earlier, workshop participants commented on accessibility and usability, and suggested amendments including reductions to the amount of text and simplifying language. Their input was essential for ensuring the appropriateness, utility and usability of the toolkit for a diverse range of stakeholders across health and social care.

Using MS PowerPoint©, the Implementation Wheel was introduced in a visual format in Workshop 1 (Figure 1) and co-production group members were asked to feedback their overall perceptions of the wheel. The design of the wheel and representation of the six implementation domains as colour coded segments (Figure 1), was positively received, with suggestions to develop the interactive capacity of domain segments as “clickable”, to enable holistic oversight of all domains with the opportunity to select specific domain(s) in accordance with implementation needs. Additionally, to increase usability options, it was also suggested that the implementation domains could be designed in a colour matched menu bar, should users prefer a more traditional route of selecting domain(s). These co-design suggestions therefore contributed importantly to the re-design and overall accessibility of the wheel from something static and one-dimensional to interactive and multi-purposeful:

*It [the implementation wheel] shows that actually all six parts of the implementation wheel are— are hugely important. That actually, you need elements of all of them in order that the implementation is going to happen and kind of bring rewards.* (Programme Manager, Secondary care, Workshop 1)

*I found it really helpful to have the domains represented visually because— especially with the colours because I tend to remember things with colours. So, it just helped me to remember what the six domains were and if they corresponded with the colours in the Word document [Implementation Checklist], I would find that really helpful.* (Academic Enterprise Researcher, University, Workshop 1)

**Figure 2:**
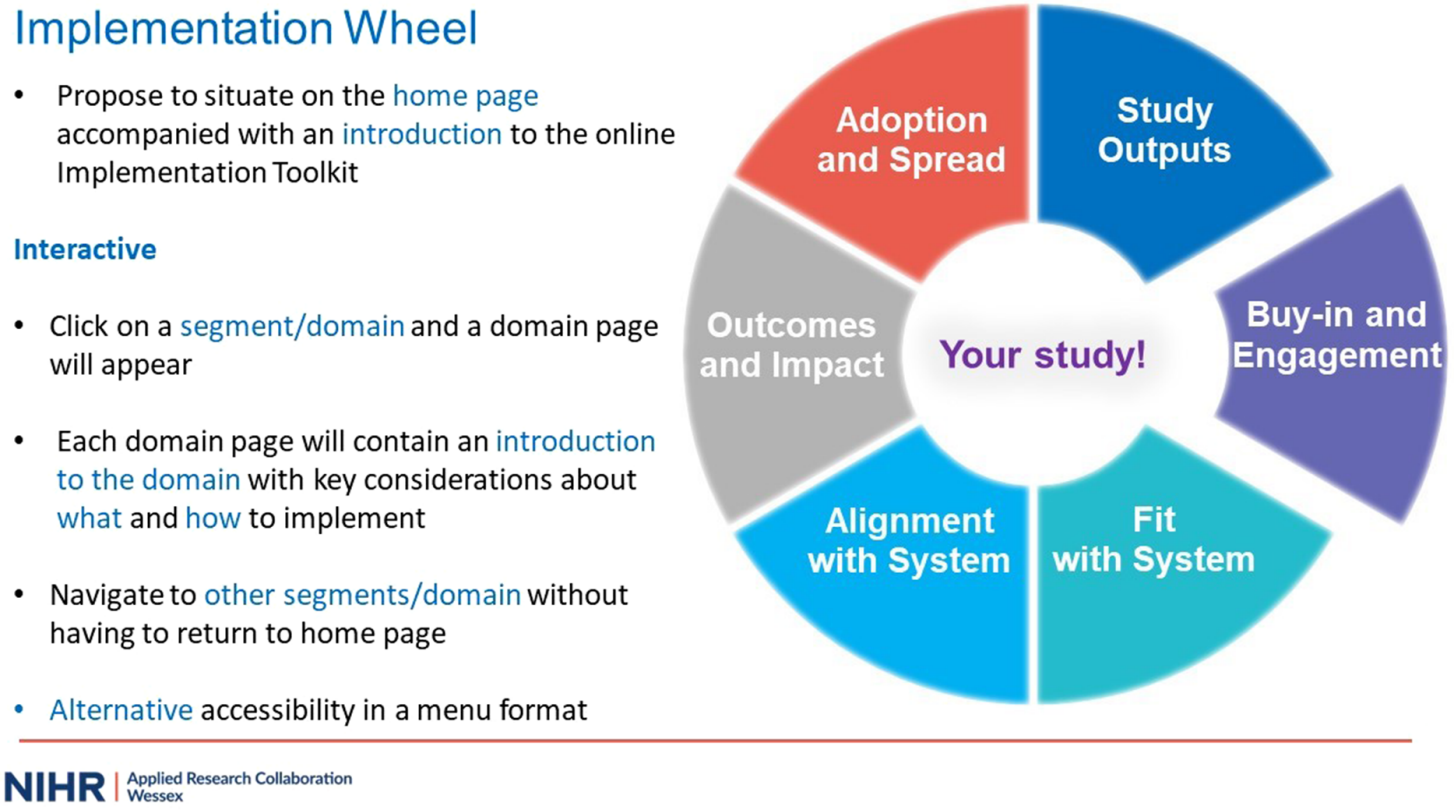
MS PowerPoint slide depicting interactive operability of the implementation wheel.

At the beginning of Workshop 2, the research team presented a summary of key discussion points from Workshop 1, suggested areas of development and subsequent actions undertaken. Feeding back to the group offered a further opportunity for clarification. With reference to design, this primarily focused upon developments to the Implementation Wheel. An updated visual representation of the wheel with instruction of interactive operability, including clickable domains, which enabled navigation to relevant domain pages was shared (Figure 2). This received positive feedback, and led to discussions as to where the wheel would best be situated in the toolkit. The consensus was that it should feature on the home page, with smaller interactive versions being available on each domain page. With this interactive capacity, the wheel was recognised not only as a key tool to support users in their implementation journey but also a navigation tool allowing users to move iteratively and flexibly across the toolkit to suit their needs:

*I really like the idea of having this [Implementation wheel] on the homepage and having the clickable sections to go in and read more about each part.* (Academic Researcher, University, Workshop 2)

*it would be great if you could hover over these things that maybe a little definition kind of just popped up because it could be that, you know, people may feel a bit more competent in those initial stages of the wheel but actually, what they're not so sure on is kind of the outcome and the impact and the adoption (…), they can kind of dip in and out of it rather than seeing it as a whole thing that they have to work through. It might just make it a bit more accessible in that context.* (Academic Researcher, University, Workshop 2)

**Figure 3:**
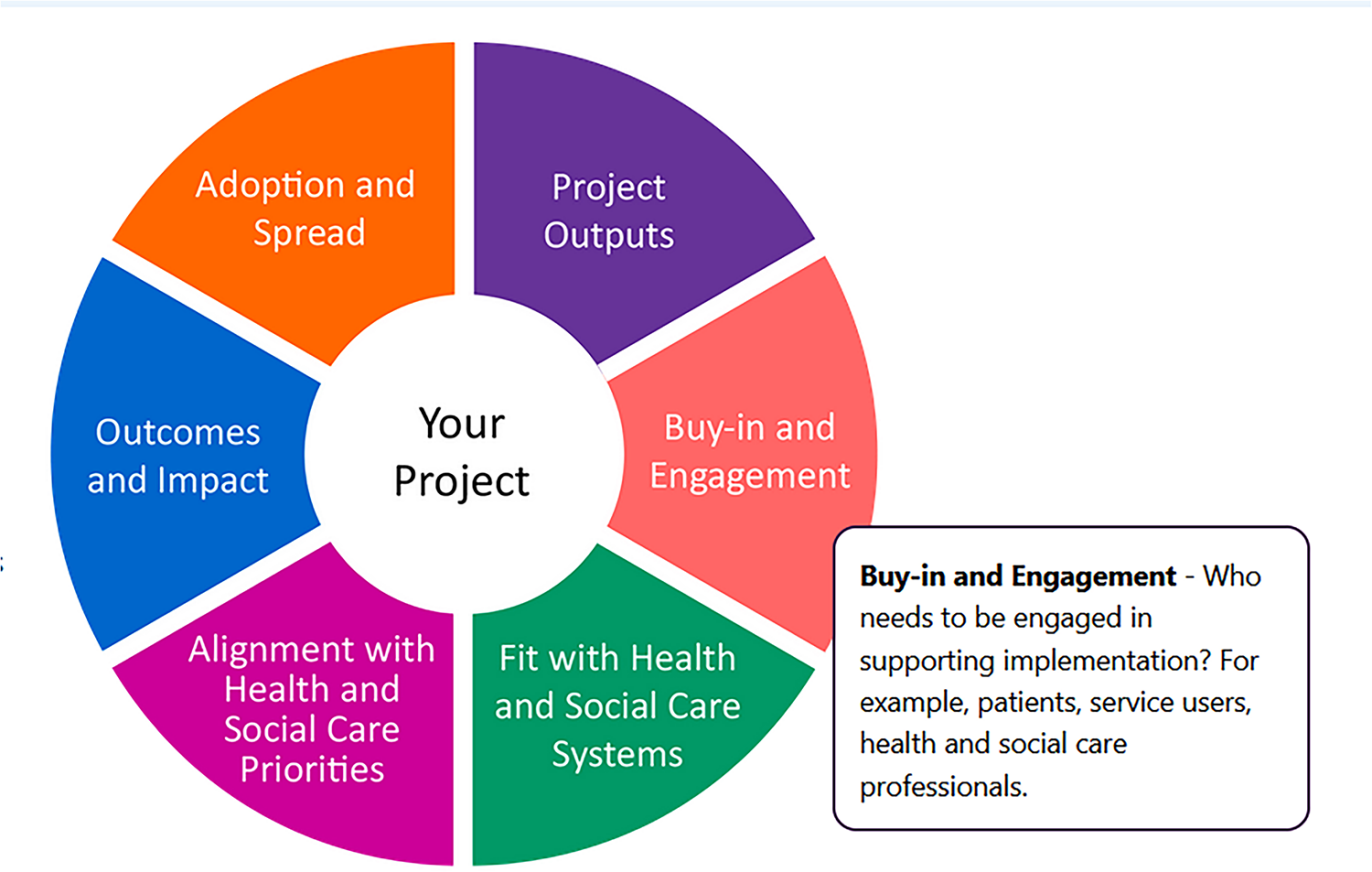
Implementation wheel on WIT home page showing pop-up definition operability.

Following the second workshop, the research team worked with the web designer to implement the amendments from the co-production group which were fundamental to the final design. Ahead of the third workshop, an interactive prototype of the home page (including the Implementation Wheel) and several of the domain pages were shared with the group who were asked to review and feedback comments on the prototype during the final workshop. For consistency and to address suggestions from Workshop 1, the research team also developed the design and format of the Implementation Checklist to match the colours of the wheel, reduce text where possible, develop considerations for users across social care and the third sector, as well as produce two versions in an editable Adobe pdf and MS Word© format to enhance utility of WIT. Domain names were also slightly amended to be more consistent with those in the Implementation Checklist. To enhance readability and contrast, colours were also enhanced by the web-designer (Figure 3).

At the final workshop, the key design developments made to the wheel described above (Figure 3), were agreed by members with additional suggestions offered:

* it looks very appealing and— and straightforward and you've succeeded really well in— in making it really nice and simple…it works well to have the wheel and then the tabs across the top* (PPI representative, Workshop 3)

*Thanks for the summary. You guys have been really busy, and I really like the look of the website. It's very usable…so, basically, there's three components. There's your implementation wheel, your implementation checklist and bitesize implementation webinars* (Academic Researcher, University, Workshop 3)

*it looks a good piece of work. It's responsive. I've actually gone onto the website both online [to] use and the laptop but also…I went on my i-Phone and it's responsive. It… it looks and feels, you know… very creative* (PPI representative, Researcher and Engineer, Secondary care, Workshop 3)

#### Co-producing a toolkit with utility for users across health and social care settings

A key outcome of co-producing was ensuring a focus on utility for users across health and social care settings, which featured as a key focal point across the workshops. As workshops progressed, co-production discussions evolved from early considerations around the purpose, intended users and name of the toolkit to more specific conversations about how to make the toolkit relevant and usable by different audiences. Suggestions resulted in amendments to toolkit functions including a drop-down interactive question and answer style of implementation considerations for each domain page as well as “pop-up” definitions of keywords to support accessibility to a wide range of users across health and social care systems.

Participants identified language as integral to enhancing the usability of the implementation toolkit. Early discussions in Workshop 1, focused upon nomenclature of the toolkit and whether it was a tool, toolkit or a resource, were viewed as important for different users and how it would be used. The word tool was identified as singular, as one component, whilst the word resource was viewed as a repository of information. The word toolkit was agreed to be most appropriate because it is a plural term comprising of constituent parts (i.e., implementation wheel, implementation checklist and implementation webinars).

*I suppose, for me— obviously, online— well, that's obvious, something online but it's— for me, a toolkit is something that's useful. It makes your life easier, something you can use to achieve what you're trying to achieve. So, the same way as a toolbox has, you know, spanners and wrenches and things that you— you can use to achieve, you know, whatever you're trying to achieve. A toolkit would be much the same. So, I want something that's flexible, that had all the tools that I needed in one place* (PPI representative and Charity lead, Third sector, Workshop 1)

*I think, the— the toolkit implies that it has— has a range of tools, a range of things that I could go away and use to consider to help answer the question that I'm looking to answer, and we've kind of eluded in this conversation the kind of range from tools of use, all the way through to self-assessment and it— there's— there's a kind of a grey scale of broadness in there as well* (Programme Manager, Secondary care, Workshop 1)

*I think, resource would mean to me something where I'm going to find out information… And, for me, the toolkit is this multifaceted thing with all the different bits in it and a tool, I would probably expect to be coming across one thing. So, that's how I would differentiate my interpretation of those words* (PPI representative, Workshop 1)

The question of intended end-users for the toolkit was also discussed. Ensuring a toolkit with relevance and utility to a range of users in health and social care settings was supported whilst there was recognition of complexity in the need to balance between providing a toolkit with value for all alongside guidance for specific users.

*I think, different areas will have their own challenges and barriers, or successes, when it comes to implementation so, I think, that— you know, rather than looking at kind of how we could solve everything… what exists already that could support people* (Charity Researcher, Third sector, Workshop 1)

*consider who the audience is for this … if you try and be everything to everybody, you might not get to a point that's useful for anybody* (Programme Manager, Secondary care, Workshop 1)

At Workshop 2, the research team presented a summary of substantive discussion points from the previous workshop, including recognition that the implementation toolkit should comprise the: Implementation Wheel, Implementation Checklist and Implementation Webinars. Regarding how the toolkit would work or be used, it was recognised that having a toolkit that was flexible and agile was essential to usability, to facilitate navigation of the complexities and uncertainties of implementation including changes to timelines, resource provision and relations between individuals and contexts.

It was suggested having keywords with “hover options,” enabling definitions to appear when “hovering” over the term, whilst not disrupting the flow of the sentence would enhance usability. User-centred language was also cited as key, with introductory sections on the main page and domain pages with direct user-centred language e.g., “you”, “outputs”, so that the user immediately perceived the website of relevance to them, regardless of their reason for accessing or occupational role. While another was to ensure that language was not limited to specific sectors (e.g., academia), so as not to exclude people from other professions, contexts or settings. In addition, co-development of a drop-down interactive question and answer style implementation considerations for each domain should be included, which users could opt to use dependent on their awareness of implementation and need:

*It would just be interesting to think about the introduction to the online toolkit like basically, an initial explanation of who this is for… I guess, something just to reassure people that they're in the right place and how this can help. Something quite brief and snappy that would just sort of encourage you to look a bit.* (Academic Researcher, University, Workshop 2)

*It would be great if you could hover over these things that maybe a little definition kind of just popped up because what they're not so sure on is kind of the outcome and the impact and the adoption… they can kind of dip in and out of it rather than seeing it as a whole thing that they have to work through … I think, to use examples, you know, — have been taken through would be a lovely way to illustrate how— illustrate how it could be used in practice.* (Academic Researcher, University, Workshop 2)

**Figure 4:**
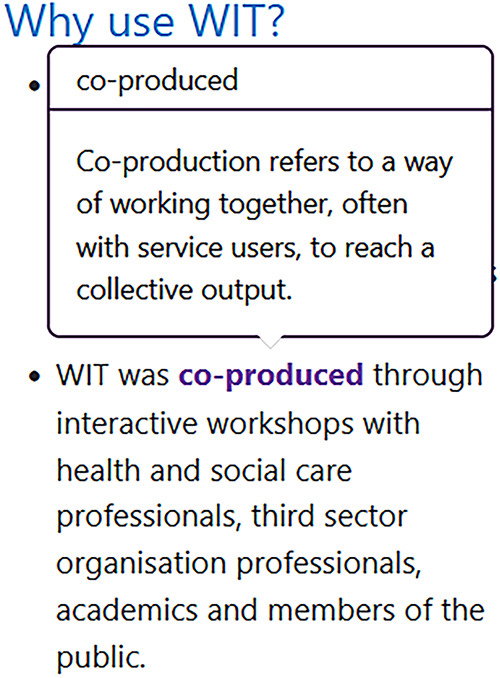
Pop-up definition of keyword function.

**Figure 5:**
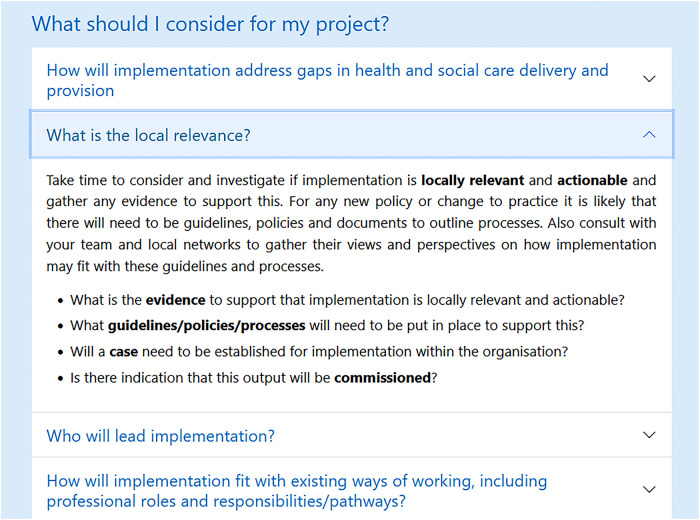
Example of an interactive drop-down question and answers on WIT domain pages.

Following Workshop 2, the research team worked with the web designer to operationalise suggestions including the incorporation of user-centred language, “pop-up” definitions of keywords (Figure 4), and interactive drop-down question and answer style implementation considerations relating to each domain (Figure 5):

An interactive prototype of the home page and several domain pages were shared with the co-production group ahead of Workshop 3. Here there was agreement that the changes made worked well. In particular the interactive drop-down question and answer style implementation considerations (Figure 5), enabling a flexible “as needed” approach to information:

*I think, when we're all time short and we're all skim-reading., I'm on the: “fit with health and social care systems” and I'm reading down the list of white coloured boxes that— they can be expanded and I really do like that you can expand each of those.* (PPI representative and Researcher and Engineer, Secondary care, Workshop 3)

*I really like the two pages that have been done on the: “fit for systems” and: “outputs”— outputs and impacts. I found it really helpful as some of you—I think, the drop-down questions just really helped bring it to life and make it feel more achievable to fill-out the checklist* (Academic Researcher, University, Workshop 3)

#### Co-producing a toolkit to support the implementation journey

The group agreed that a key function of the toolkit was to support users on their implementation journey. Early discussions focused upon the need for an agile toolkit to enable this and suggested providing examples of implementation challenges. For example, creation of additional work for those involved in implementation or not having time or resources to support implementation. Discussions led to examples that were co-produced and included challenges and solutions operationalised within the “question and answer” style drop-down menus on the domain pages.

The need for an agile toolkit was recognised early in the co-production process, as a way for users to flexibly navigate the complexities and uncertainties of implementation including changes to timelines, resource provision and relations between individuals and contexts. Group members offered examples of these across different health and social care contexts:

*To help guide me at all the different stages at which I might be thinking about implementation and it might be that that gets split into different parts of the process….I guess, I would want it to highlight to me solutions to the kinds of common problems that we face at the stage— the stage of implementation… And there are bound to be other sort of common problems that people are facing that your expertise could help guide— give guidance on and perhaps sort of examples of how other people have done it. Sort of stories, modelling, how other projects did things might be useful.* (Academic Researcher, University, Workshop 1)

*So, you almost create a journey, don't you, through that kind of implementation for that research and those involved in that.* (Charity Researcher, Third sector, Workshop 1)

The co-production process enabled examples of challenges and solutions to be shared during workshops which enhanced discussions about adoption and sustainability of the toolkit following its development. For example, proposals put forward included involving users and beneficiaries in the planning for the study to maintain longer-term impact, considering relevant evidence needed to persuade stakeholders, and resource considerations beyond the life cycle of the project. The group also reiterated the importance of flexibility in addressing these, to accommodate potential challenges:

*So, any implementation of any project is not linear, it comes and goes, there's different waves, there's different points in time, there's things that are in your control and out of your control. So, I think, having implementation plans that are flexible and then can adjust and accommodate all those different things throughout your implementation period, is actually really important* (Programme Manager, Secondary care, Workshop 2)

*So, I say that in terms of timing and priorities within organisations so, you know, from a— so, a sector perspective as we enter a new strategy, we've got strategic priorities out— which might fall outside of those or they may not be relevant no more so, therefore it may be really great, we may have all the intention to kind of implement that but actually, we can't because it's not part of our strategic direction or way that we want to go. So, I think, there's that consideration of kind of things that kind of— we can't control and actually, it's more meaningful that we don't try and shoehorn something (laughing) into a space and it be used meaningfully.* (Charity Researcher, Third sector, Workshop 2)

Following the workshop, the research team worked with the web designer to incorporate these examples within the interactive “question and answer” style drop-down options (Figure 5), and case studies to depict navigation of these considerations in projects across health and social care. These examples and case studies were included as part of the prototype implementation toolkit shared ahead of Workshop 3. Due to their embedded nature within the context of relevant implementation questions and answers, the examples, were felt to have more utility than the case studies which were longer and in narrative form. Instead, the case studies were considered a useful endorsement of how to use the toolkit to support a specific project:

*I favoured having examples so that when you click-on your drop-down and you're looking at the thing, to actually have a few examples… That's really valuable* (PPI representative, Workshop 3)

*having the specific examples within each drop-down is actually really helpful because then you're looking at it— you've got an example at the same time as you're reading the definition.* (Academic Researcher, University, Workshop 3)

*I guess, the case studies are almost…more of an endorsement of like the tool and so they could almost be like collated- it's almost evidence, isn't it?* (Academic Researcher, University, Workshop 3)

## Discussion

In this paper, we have reported findings from a study to co-produce a web-based implementation toolkit (WIT) to facilitate adaptive implementation across health and social care systems and have shown the value of a co-production approach to toolkit development. The co-production group, involved a diverse range of end-users across different settings who through participating in a series of online workshops which provided space for reflection, discussion and refinement, worked in partnership with researchers to co-develop an accessible and usable toolkit to support the implementation of changes to practice or innovations in health and social care systems. The benefits of co-production enabled support for the translation of implementation into health and social care systems to improve outcomes for a variety of people. The value of co-production is demonstrated through three core themes:

• Enhancing accessibility and usability through design
• Relevance of implementation for a variety of users across health and social care settings
• Supporting adaptive implementation in accordance with user needs

### Enhancing accessibility and usability through design

Including those involved in implementation or, in the case of PPI representatives, as service users through co-production ensured style, images, diagrams and colours that are important to end users were included in a way that increased the accessibility and usability of WIT. Drawing upon the example of the Implementation Wheel, a core component of WIT, we demonstrate how involving stakeholders in shared decision-making informed co-development of the wheel from a visual static image through to an interactive online tool, with further functionality to navigate across WIT as a whole. The value of co-production processes with a variety of stakeholders to increase the accessibility and usability of toolkit uptake, strongly resonates with other research advocating iterative co-production methods to develop interventions with stakeholders (28–30).

Co-production of WIT through online workshops incorporating iterative cycles of consultation, reflection and feedback addresses the call for more qualitative and pragmatic approaches within both implementation science and co-production work (22). It extends opportunities for exploration of complex concepts and inclusion of a variety of stakeholder views (31). For example, the co-development of a hover “pop-up” definition function of key implementation terminology across WIT suggested by the group, increases the inclusivity of WIT for people who may have otherwise been unfamiliar with the terminology, concepts or relevance to practice. The PPI representatives in the group were invaluable in this respect by challenging any assumptions of knowledge those more familiar with implementation had and challenging use of terminology that was not transparent for all.

### Relevance of implementation for a variety of users across health and social care settings

Co-production with stakeholders across health and social care systems, serves to increase awareness of the value of implementation for users across these systems. In our study it promoted interdisciplinary knowledge exchange and learning, raising awareness of considerations specific to different perspectives and contexts (32). Participants continually brought to the fore the importance of considering the relevance of WIT to the end user. For example, through initial discussions around the value of WIT and consensus to describe WIT as a “toolkit” rather than as a tool or resource, to later discussions, surrounding practical challenges and enablers participants had encountered in their experience of implementation across health and social care systems, which were embedded in interactive drop-down “question and answer” style menus on the domain pages.

The co-production group worked well together and showed mutual respect in terms of listening, acknowledging and supporting different views and perspectives during the WIT development process which helped to support this collaborative approach. In this sense, WIT is an example of successful co-production. Though the group was diverse in terms of roles and representation, consensus was achieved and where differences of opinion these were resolved satisfactorily for the group. However, research suggests that our experience is not necessarily the norm, and has highlighted potential barriers of competing priorities and interdisciplinary conflict between stakeholders across different fields when working together (27, 28). We suggest that the collaborative environment established in the co-production workshops, may also have been underpinned by a reiteration of the overall shared purpose of the project at the beginning of each workshop and the importance of co-producing a toolkit that had utility and value for a variety of users throughout the workshops. Also, the key role played by PPI participation, which ensured the group remained focussed on the key aims and were not distracted or diverted when other issues or professional agendas could have diverted the focus of the group.

### Supporting adaptive implementation in accordance with user needs

Adopting a co-production approach to the development of WIT, whereby stakeholders from a range of roles with differing implementation experience and knowledge, across health and social care systems, enabled rich and meaningful data to be generated that informed the development of an agile and flexible toolkit, to guide users on their implementation journey. The sharing of their own experiences of implementing complex interventions in health and/or social care, or as a recipient of the intervention demonstrated the complexity of implementation, and the relational dynamics between individuals, local contexts and wider health and social care systems and implementation challenges and enablers, which were included as examples and case studies in the toolkit. In doing so, as co-designers of the toolkit, through their contributions, participants encouraged holistic oversight of these interactions and “normalised” implementation challenges, supporting and encouraging users in navigating adaptive and complex situations, such as those compounded by Covid-19 (1–5, 33).

### Online co-production processes as a mechanism for encouraging collaboration

Reflecting upon the co-production processes involved in the online workshops, also contributes to informing co-productive practices, and an opportunity to consider what works well and what does not (22). In our experience online co-production workshops, with activities to elicit reflection, discussion and refinement offered opportunities for enhanced co-production and inclusivity. Firstly, online workshops, did not involve the Covid-19 infection risks associated with travelling to or attending in-person workshops. Secondly, through virtual participation, they negated time, travel and related expenditure considerations more generally (34, 35); enabling a cross-section of stakeholders from a wider geography to participate at shorter notice (36). The “chat” function on Zoom was useful because it gave an alternative opportunity for people to contribute without having to verbalise their comments and also enabled inclusion of those who were less confident to speak less in a group. The “raise a hand” function helped with turn taking, giving an opportunity for participants to signal their intention to speak to the facilitators. Both these functions provided additional options for participation thereby enhancing inclusivity. This resonates with other work which highlights the benefits of online modalities in terms of representation, inclusivity and accessibility, whilst acknowledging potential limitations, including information technology literacy and accessibility, including internet access and observing body language cues (36).

It is worth noting that participation in online co-production workshops requires a number of accessibility and usability considerations which may not be available to all. For example, in this study, PPI representatives were recruited from established public and patient involvement networks where participation in online and in-person research workshops may be more familiar and therefore may not be representative of PPI contributors who had not been recruited from these sources. PPI representatives also had good and reliable internet access, and ensuring adequate costings in the funding budget for PPI enabled patient and public contributors to be fully reimbursed for their participation in workshops and as co-authors on publications. The opportunity to build language translation functionalities into WIT may further improve accessibility and may lead to increased engagement.

## Conclusion

Co-production provides unique opportunities for interdisciplinary knowledge exchange and learning, increasing awareness of implementation considerations and its importance in translation of outputs into practice. By embedding stakeholder experiences of implementation within WIT, it highlights the complex relational dynamics between users and health and social care systems to provide a flexible and agile toolkit to support users on their implementation journey. Co-production of WIT with a variety of end-users across health and social care enhances the utility, accessibility and appropriateness of WIT and the translation of implementation across these settings to benefit outcomes for a variety of people.

## Data Availability

The data and materials are not available for open access, due to restrictions governed by the ethical agreement approved by the University of Southampton. Requests to access the datasets should be directed to c.f.brooks@soton.ac.uk.
